# Forest waste composting—operational management, environmental impacts, and application

**DOI:** 10.1007/s11356-024-32279-0

**Published:** 2024-02-19

**Authors:** Maria Elisabete Ferreira Silva, Raffaella Saetta, Roberta Raimondo, José Manuel Costa, José Vicente Ferreira, Isabel Brás

**Affiliations:** 1 CISeD-Centre for Research in Digital Services, Polytechnic Institute of Viseu, 3504-510 Viseu, Portugal; 2 LEPABE-Laboratory for Process Engineering, Environment, Biotechnology and Energy, Faculty of Engineering, University of Porto (FEUP), R. Dr. Roberto Frias S/N, 4200-465 Porto, Portugal; 3 Department of Civil, Building and Environmental Engineering, University Napoli Federico II, Via Claudio, 21, 80125 Naples, Italy; 4 Research Center for Natural Resources, Environment and Society (CERNAS), Polytechnic Institute of Viseu, 3504-510 Viseu, Portugal

**Keywords:** Composting, Life cycle assessment, Forest fires, Forest and agriculture residues, Residual Biomass Collection Centre, Soil

## Abstract

In Portugal, the number of fires and the size of burnt areas are rising dramatically every year, increasing with improper management of agroforestry wastes (AFRs). This work aims to study the composting of these wastes with minimal operational costs and understand the environmental impact and the compost application on burnt soil. Thus, a study of life cycle assessment (LCA) was carried out based on windrow composting processes, considering the avoided environmental impacts associated with the end-product quality and its application as an organic amendment. Three composting piles were made with AFRs from the Residual Biomass Collection Centre (RBCC) in Bodiosa (Portugal). Sewage sludges (SS) from an urban wastewater treatment plant were used as conditioning agent. One pile with AFRs (MC) and another with AFRs and SS (MCS) were managed according to good composting practices. Another pile with the AFRs was developed without management (NMC), thus with a minimal operational cost. Periodically, it was measured several physical and chemical parameters according to standard methodologies. Eleven environmental impacts of compost production, MC and MCS, were analyzed by a LCA tool, and their effect on the growth of *Pinus pinea* was evaluated, using peat as reference. Composting evolution was expected for both piles. Final composts, MC and MCS, were similar, complying with organic amendment quality parameters. Compost NMC, with no operational management, showed the highest germination index. Piles MC and MCS showed similar environmental impacts, contributing to a negative impact on global warming, acidification, and eutrophication. Greater growth was obtained with application of MCS, followed by MC, and finally, peat. Composting is a sustainable way to valorize AFRs wastes, producing compost that could restore burnt soils and promote plant growth and circular economy.

**Graphical Abstract:**

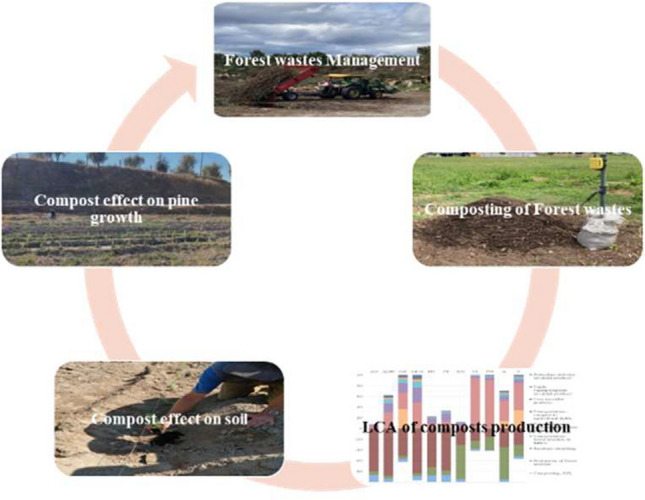

## Introduction

Portuguese territory occupies a strategic position for forest growth, covering about 35% of the territory (Da Costa et al. 2020). However, the number of wildfires and the size of burnt areas are rising dramatically every year (Ferreira-Leite et al. 2011). A direct consequence of the increase in forest fires is the deterioration of the burnt soils. Changes in climate and fire regime reduce ecosystem services by increasing soil degradation and losses in plant diversity. High burn severity limits natural vegetation recovery and reduces both biological and soil functionality (Meira Castro et al. 2020).

Ash and soil erosion, as well as nutrient losses, are very significant when intense rainfall (typically attended by powerful winds) occurs in the period immediately after a fire. During this time, soils are very sensitive to any kind of disturbance, especially because vegetation has not yet started to recover. The lack of soil protection and the sparse ash cover increase the impacts of raindrops on soil compaction and facilitate sediment detachment. These conditions are very common in Mediterranean environments because of the occurrence of high-intensity and short-duration summer thunderstorms and intense autumn rainfalls (Shakesby and Doerr 2011; Certini 2005). Moreover, it is urgent to implement strategies of forest management that reduce wildfires and promote soil restoration. Therefore, increasing attention is devoted to soil management practices aimed to improve both soil organic carbon incorporation and soil organic matter (SOM) accumulation, as the addition of organic materials and, even more, the use of compost obtained by facilities located in agricultural companies (Pergola et al 2020).

In Portugal, only 3% of forest areas is kept by the State and 12% is ownerless. Therefore, 85% of these areas, with an average size of 5 ha, belong to about half a million owners, often elderly, also considering the demography in rural areas (Gomes 2006). As a result, it is difficult to implement consistent and effective management of agroforestry residue (AFRs) practices to reduce wildfire risks.

To improve the AFRs management, strategic Residual Biomass Collection Centres (RBCC) may be developed, as public sites where all citizens can deliver the residual biomass waste resulting from pruning and cleaning of agriculture, forest, or gardens. AFRs are all tree’s components, including bark, needles, roots, leaves, branches, and trunk as well as agricultural residues collected by landowners. All these materials may take part in the circular bioeconomy, a late-developing concept defined by Carus and Dammer (2018) as the intersection of the bioeconomy and the circular economy to involve the use of biomass in a sustainable way, with the efficient valorization of biomass resources within the production chain. The wastes from these RBCC are often used for electrical energy production in a local biomass powerplant. However, the valorization of AFRs through composting, to produce an organic soil amendment that improves the quality of burned soils, reducing the use of chemical fertilizers, may arise as a valuable practice considering the overall life cycle of the biomass.

The eco-sustainable AFRs valorization process by composting is still not very widespread in Portugal: few studies were carried out with practical application, and generally, these studies deal with urban or agricultural waste composting. Life cycle assessment (LCA) is an approach to evaluate the environmental aspects and potential impacts associated with a product, process, or service throughout its life cycle (Ferreira et al. 2021a, b). In processes like composting under different conditions, LCA helps identify and quantify its associated environmental impacts, namely, the associated resource depletion, energy consumption, greenhouse gas emissions, water usage, toxic emissions, and other environmental stressors, helping the decision-makers about the most environmentally friendly process and the one to be followed.

According to Oviedo-Ocaña et al. (2023) in their systematically review of LCA studies in biowaste and/or green waste composting, the avoided environmental impacts associated with the end-product quality and its application as an organic amendment or soil improver were ignored. Moreover, the LCA studies related to green waste composting are scarce. LCA studies rarely report the quality of the processed waste, most of the studies only specified the amount of waste to be processed and its main composition (Oviedo-Ocaña et al 2023). These studies show that the environmental impacts of composting are very sensitive to compost facility management practices for maintaining aerobic conditions (e.g., the technology used and operation conditions).

Despite the environmental benefits, composting could have negative direct and indirect environmental impacts. Direct negative impacts are related to CO_2_ emissions from decomposing organic matter in composting system and that usually are not considered as additional greenhouse gases (GHG) emissions since they are biogenic and part of the short-term C cycle (Brown et al. 2008). Moreover, there are methane (CH_4_), nitrous oxide (N_2_O), and ammonia (NH_3_) emissions from methanogenic and denitrification processes occurring during the composting process under anaerobic conditions, which result in odor and additional GHG emissions (Saer et al. 2013; Oliveira et al. 2016).

Serafini et al. (2023) reported in their review work that the most cited environmental impacts were global warming potential, acidification potential, eutrophication potential, photochemical oxidation potential, and ozone layer depletion, as gaseous emissions from the transport and decomposition represent the main contributors to these categories. Composting environmental impacts were also highly related to the use of non-renewable energy sources. Therefore, home composting was considered the one of the best environmental options (Serafini et al. 2023). Therefore, it is critical to evaluate the impacts of composting using sustainability assessment methods and tools (Weligama Thuppahige et al. 2022).

This work aims to study if composting of agroforestry wastes (AFRs) may contribute to restore the burnt soil increasing the forest growth, with a minimum environmental impact and minimal operational cost. Thus, a study of life cycle assessment was carried out based on windrow composting processes, considering the avoided environmental impacts associated with the end-product quality and its application as an organic amendment.

## Methodology

The study was conducted in one of the RBCCs in the municipality of Viseu (Portugal), located in the parish council of Bodiosa. The RBCC of Bodiosa is operational since 2019, with an area of approximately 5000 m^2^ and a rectangular geometry. The residual biomass that is allowed to be deposited at the RBCCs belongs to two categories: forest residues (FR) from forest management (stumps, roots, leaves, and branches) and agricultural residues (AR) from agricultural activities and the agrifood industry, including residues from cereals, rice, orchards, olive groves, and vineyards. In the RBCC under study, both local FR and AR are stored, and this work leans over a mix of these two types of residual biomass, the agroforestry residues (AFRs).

### Composting processes

The composting process was done to the shredded residual biomass (AFRs) and it was carried out with 3 cone-shaped composting piles (2-m base diameter and 1.2–1.5-m height) (windrow system). The 3 piles were AFRs with periodic manual turning and irrigation (MC); AFRs with addition of sewage sludge (SS), with periodic manual turning and irrigation (MCS); and AFRs with no interventions (NMC). The AFRs shredded had a density of 243 kg/m^3^. For the MC and NMC piles, 828 kg of biomass were used, corresponding to a total of 3.4 m^3^ of AFRs. The preparation of the MCS pile was done with 1.88 m^3^ of AFRs and 0.68 m^3^ of SS. The SS used was from a local wastewater treatment plant. The pile turning was carried out with the help of a rake: since handling is manual, no material or energy is consumed. The moisture content was adjusted whenever it fell below 45%. The correction was made four times during the process evolution by adding water, with a hose and a watering can.

The management of MC and MCS piles was done by daily measuring of the temperature and on a weekly basis the measuring of moisture, electrical conductivity (EC), pH, total organic matter (TOM), ashes, total organic carbon (TOC), and total Kjeldahl nitrogen (TKN). When the pile temperature decreased to near ambient temperature (Amb), these parameters were evaluated monthly. The process was completed on day 120. Sample collection for laboratory analysis was done at different heights and depths, in order to triplicate the measurements. To evaluate the compost quality, physical, chemical, and biological analyses were carried out as indicated in the Ordinance nº185/2022 (2022) for corrective organic fertilizers. The standard procedures used for analytical control were EN 12580 for Bulk density; EN 13038 for EC; EN 13037 for pH; EN 13040 for Moisture; EN 13040 for TOM and Ashes content; EN 13654 for TKN; EN 13652 for nitrates (NO_3_^−^-N); EN 16086 for Phytotoxicity; and EN 13650 for Nutrients and heavy metals. The maturity level through the self-heating process was carried out according to Gutesichererung Kompost RAL-GZ 251. The TOC was achieved by dividing TOM by 1.8 (Jiménez and Garcia 1992).

### LCA study

The life cycle assessment of the composting processes was carried out according to ISO 14040: 2006 (ISO 2006a), ISO 14044: 2006 (ISO 2006b), and the International Reference Life Cycle Data System (ILCD) (European Commission 2010). The LCA procedure is structured in four steps: goal and scope definition, life cycle inventory (LCI), life cycle impact assessment (LCIA), and interpretation and presentation of results. To accomplish the LCA, the software *SimaPro 9.3.0.3 PhD* was used.

### Goal and scope definition

**Fig. 1:**
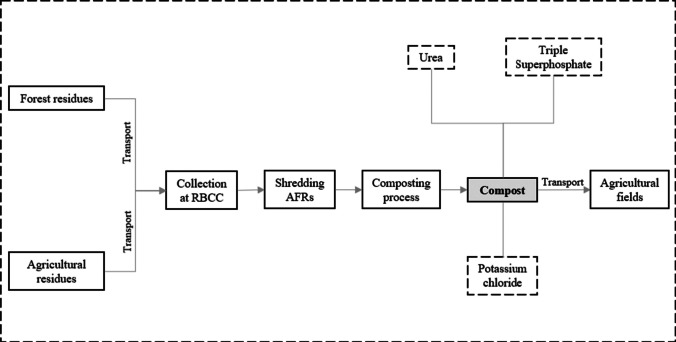
Definition of the system boundaries in the AFRs composting process (Windrow system)

LCA study goal is to assess the environmental impact of compost production from MC and MCS piles, according to a cradle-to-grave study. The functional unit (FU) of the study is 1 tonne of AFRs composting at the RBCC of Bodiosa. The study limits begin with the production of the AFRs and transportation to the RBCC, where the shredding is performed. The composting process includes biomass cone-shaped piles construction, irrigation, if needed, and manual handling, biological composting process during 120 days, transportation of the compost to agricultural fields, and their manual distribution for application, where it is used as an organic soil conditioner (Fig. 1).

All the input and output loads are referred to the functional unit of the study. The composts obtained have an economic and environmental value, hence an impact on market dynamics, concerning with the demand for conventionally used chemical fertilizers. Consequential approach in conducting the LCA was used to consider the avoided production of such goods.

For both cases, the quantity of fertilizer replaced by compost is assessed on the basis of their nutrient content, and the final destination of the nutrients that are not absorbed by plants is also considered in the study, since they represent an environmental burden. The influence of the obtained products on the carbon cycle, downstream of their distribution, is excluded from the system boundaries due to the complexity of the phenomena.

### Life cycle inventory (LCI)

#### Arising of the AFRs

According to Ferreira et al. (2021a, b), the impacts associated with the production of forest residues are mainly explained by the impacts of maritime pine’s (*Pinus pinaster*, *Aiton*) process and pruning, considering this one of the most representative wastes in the RBCC. Data presented by these authors was the reference for the present analysis.

#### Transport and shredding

It has been defined a segment of the area from which users can travel with an agricultural vehicle should be less than 10 km on the OpenStreetMap road network to reach the RBCC. According with the AFRs characterization (Brás et al. 2023), the biomass in the RBCC is mainly forest wastes, and it was considered to be 0.76 ton of forest wastes, per functional unit. These wastes arise of larger areas of the territory, comparing with the agriculture, and therefore require more time and fuel to be picked, with a greater impact in terms of transport. The overall estimated time was considered the amount to be transported, the time required, and the truck capacity.

The biomass shredding for further use was done by an excavator with a grapple and was fed into a crushing equipment with a hammer mill. The machinery productivity is 20 ton/h. Three minutes are required to crush 1 tonne, which is the operating time of both machines. There is a 2% loss of dry matter in the biomass as a result of shredding (Forsberg 2000; Whittaker et al. 2016). The data is shown in Table 1.

**Table 1:** Inventory for the AFRs transport and shredding

Inventory		Process/material	Quantity	Unit measure
Transport	Inputs	Maritime pine wood, at forest road ^1^	1.39	m^3^
		Transportation of forest residual biomass	7.48	min
		Transportation of agricultural residual biomass	2.42	min
	Outputs	Forest wastes, at RBCC	0.76	ton
		Agricultural wastes, at RBCC	0.24	ton
Shredding	Inputs	Wastes, at RBCC	1	ton
		Biomass shredding	3	min
	Outputs	Shredded biomass, at RBCC	0.98	ton

^1^Ferreira et al. (2021a)

#### Composting

For the LCA studies, only the monitoring piles were compared (MC and MCS). Since no measurements are available for the NMC pile, it can be assumed that the compost impact is the same as for MC pile, due to their similar characteristics. From the direct measurement of the pile’s dimensions at the end of the process, the final volume has been evaluated. To assess the final weight of the piles, compost density values were taken from the laboratory analysis carried out in previous stages of the work (data not published). Table 2 shows the values obtained for the MC and MCS piles.

**Table 2:** Physical characteristics of the piles, before and after the composting process

Parameters	MC		MCS		
	Raw material—AFRs	Compost	Raw material—AFRs	Raw material—sludges	Compost
*d* [kg/m^3^]	243.5	397.2	243.5	1800	457.9
*V* [m^3^]	3.40	1.31	1.88	0.68	1.82
*m* [kg]	827.8	518.5	458.9	1221	835.2
***I***_**C**_** [-]**	**63%**		**50%**		

A compost production index, *I*_c_, was assessed to know the amount of compost produced. The volume of the piles and the density of the materials were known; thus, the mass of the piles was estimated.

Since emissions were not monitored during the composting processes, it was considered the results from literature, ADEME (2015) and Ecoinvent (2021). Compost is transported by agricultural vehicles to the fields, to be used by local farmers as a soil conditioner. To consider the transport-related impacts of the only quantity of compost produced from FU, the load factor of the vehicle was estimated, as the ratio between the mass of compost transported and the capacity of the vehicle (assumed to be 1 tonne). It was multiplied by the load factor to estimate the proper time parameter. From the compost nutrients composition, the amount of avoided fertilizers production was estimated, namely, urea, potassium chloride (KCl), and triple superphosphate (TSP). The equivalence in terms of nutrients between compost and mineral fertilizers was established based on the respective nutrients’ bioavailability. The amount of N contained in urea is 450 g/kg and 56% is available for plants. Regarding TSP, its P content is 210 g/kg, of which 86.5% is available for plants (Mullins and Sikora 1994). The amount of K in the KCl is 498 g/kg, and 67.5% of this K is available for plants (Naylor and Schmidt 1986). The percentage of total phosphorus contained in the piles of compost (in dry mass) is 0.24% (Llonch et al. 2021). The nutrients in the compost are supposed to be fully bioavailable to plant species, resulting in a lack of emissions due to unabsorbed ones. If compost is stable, an essential requirement for its use in agriculture, it generates minimal emissions after its application. Also neglected, as they are difficult to quantify, are the benefits generated by compost after its use. In Table 3, input and output data for the two piles are shown.

**Table 3:** Inventory for the composting processes

	Process/material	MC	MCS	Unit measure
Inputs	Shredded biomass	0.980	0.980	ton
	Oxygen	267.000	213.000	kg
	Land use	14.172	5.540	m^2^/year
	Water	0.437	0.215	m^3^
	Transportation to fields	6.365	5.051	min
Outputs	Compost	0.614	0.487	ton
	NH_3_	0.020	0.016	kg
	CO_2_	404	320.000	kg
	N_2_O	0.051	0.040	kg
	CH_4_	0.047	0.037	kg
	NMVOC	0.019	0.015	kg
	H_2_O	0.189	0.150	m^3^
Avoided products	Urea	10.650	12.830	kg
	TSP	3.176	2.521	kg
	KCl	5.487	3.234	kg

*NMVOC*, non-methane volatile organic compound; *TSP*, triple superphosphate.

According with the consequential approach, considering the amount of N, P, K available for plants, the amount of avoided fertilizers was assessed.

### Life cycle impact assessment (LCIA)

The CML-IA baseline V3.07/World 2000, a midpoint approach, was selected as the impact assessment method. The results can be normalized, but neither weighting nor summation is provided. The impact categories considered were abiotic depletion (AD), abiotic depletion associated to fossil fuels (ADff), global warming (GW), ozone layer depletion (OLD), human toxicity (HT), fresh-water aquatic eco-toxicity (FE), marine ecotoxicity (ME), terrestrial ecotoxicity (TE), photochemical oxidation (PO), acidification (A), and eutrophication (E).

### Plant growth studies

A growth trial was set up on stone pine (*Pinus pinea*). Seventy-two pine trees averaging 17 cm were planted and tested in two water regimes and three fertilizers. After an initial conditioning/watering of all the plants, 36 pine trees were planted and water-stressed and the rest were irrigated (30 mm of water per week). Three fertilizers were tested for each water regime. After tilling the soil, around 500 cm^3^ of each of the fertilizers, MC, MCS, and peat (P), were placed in the planting holes, with 12 replicates each. Plant height was measured fortnightly during the first 5 months and three more times in the 2nd year of the trial. The measurements were done vertically, from the plant’s insertion into the ground to the last apical bud (excluding the stems).

Chlorophyll fluorescence measurements using chlorophyll fluorometer (Handy PEA +) were performed on all stone pines from each trial after 1 year of the experiment. Maximum Photosystem II (PS II) activity was calculated considering the *F*_v_/*F*_m_, that is, presented as a ratio of variable fluorescence (*F*_v_) over the maximum fluorescence value (*F*_m_), considering *F*_o_ as the zero fluorescence level, according with Eq. 1 (Maxwell and Johnson 2000).

1 $$\frac{{F}_{{\text{v}}}}{{F}_{{\text{m}}}}= \frac{({F}_{{\text{m}}}-{F}_{{\text{o}}})}{{F}_{{\text{m}}}}$$

Soil was also characterized, namely, by the cation exchange capacity (CEC), using the ammonium acetate (pH 7) method (Sumner and Miller 1996). Total organic matter was determined following the volatile compounds loss on ignition (LOI) (Gee and Bauder 1986) after ashing samples at 400 °C for 8 h. Soil pH was measured in a soil suspension prepared as a 1:1 soil to water ratio using an Accumet XL20 pH/conductivity meter (Fisher Scientific, Pittsburgh, PA, USA) (United States Department of Agriculture, Natural Resources Conservation Service 1995).

## Results and discussion

### Composting process

The AFRs are mostly characterized by forest, agricultural, and pruning residues. In Table 4, the physical and chemical characteristics of AFRs and SS used for the composting process are reported. The main differences between these two wastes are the bulk density, moisture content, minerals (as seen by the electrical conductivity of the materials) and pH. Overall, the SS showed the highest content for these parameters, and they were according with those found in the literature (Asses et al 2018; Guo et al 2020; Zorpas and Loizidou 2008).

**Table 4:** AFRs and SS initial characterization

Parameters	AFRs	SS
Bulk density [ton/m^3^]	0.243 ± 0.001	1.800 ± 0.001
Moisture [%]	42.5 ± 1.0	81.5 ± 0.9
TOM [%]	72.5 ± 0.4	71.8 ± 0.9
Ashes [%_dry mass_]	24.6 ± 4.1	28.2 ± 0.9
TOC [%_dry mass_]	40.3 ± 0.2	39.9 ± 0.5
TKN [%_dry mass_]	1.32 ± 0.05	3.38 ± 0.02
pH	5.51 ± 0.05	7.20 ± 0.02
EC [mS/cm]	1.59 ± 0.08	1.80 ± 0.02

Several parameters were monitored periodically during the 120 days of the composting process to study the evolution of MC and MCS piles. Moisture is very important since the relative proportions of water and dry matter can control the biological activity, responsible for the degradation of total organic matter (TOM), and the aerobic conditions required for the process to take place. Also, TOM is a key factor once compost has a major role in soil amendment and therefore is fundamental to assess the organic supply added to the soil, to restore the TOM content in soils, mitigating the desertification and the erosion phenomena. TOM is directly related to total organic carbon (TOC) which points out the most stable and slow-release fraction of organic carbon. Nevertheless, temperature is one of the main parameters for monitoring the composting process (Xiu-lan et al. 2016; Raut et al. 2008), and it is also a function of the process (Turan 2008). Composting is an exothermic process, and temperature rises because of the accelerated biodegradation of organic matter by microorganisms (Raut et al. 2008). Temperature has a significant role in the microbial dynamics during composting, and its study allows us to understand the process evolution through the different composting stages (mesophilic, thermophilic, cooling, and maturation).

**Fig. 2:**
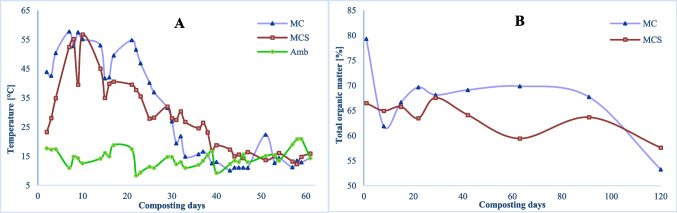
Evolution of the composting processes: **A** temperature profile; **B** TOM evolution

Figure 2A shows the temperature profile of the 2 monitored piles and the ambient temperature (Amb). MC pile reaches a maximum temperature of 57.7 °C on the 7th day of composting; indeed, in the early stage of the process, there is a rapid reduction of TOM (Fig. 2B). A further peak of 54.8 °C is reached after 21 days of composting. The pile MC only with AFRs reaches a thermophilic stage earlier than the pile MCS with a mix of AFRs and SS. After 40 days of composting, the average temperature of the piles was around ambient temperature.

**Fig. 3:**
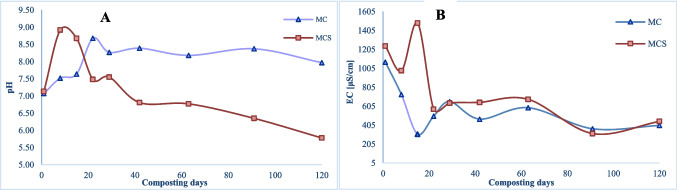
Parameters evolution in the composting process: **A** pH; **B** electric conductivity (EC)

It is expected that organic matter decreases during the composting process because it is used up by microorganisms as an energy source for metabolic activities. Studying the evolution of organic matter over time allows distinguishing the various stages of composting, which are characterized by different degradation rates of organic matter. On the other hand, this also allows us to understand whether the amount of air present in the interspaces is adequate for microorganisms’ activities, suggesting the best periods for turning the pile. The piles were turned every 3 days during active degradation, then weekly until the temperature reached the ambient temperature. In MC pile, the TOM profile showed a high decrease in the beginning and did not decrease significantly over the subsequent 60 days, but in the last phase, between 90 and 120 days, 14% of TOM have been consumed, achieving an overall rate of 19.3% consumption. It was verified that lower TOM consumption in MCS results in a degradation rate of 14.2%, but in this pile, it was verified a uniform decay. However, this difference between the two piles must be related to the raw materials used. In the MCS was added SS, whose TOM is more stable and less biodegradable by microorganisms. Besides, it should be considered that the pH (Table 1 and Fig. 3A) of MCS was kept acidic from the cooling process until the end of composting. That might have caused adverse conditions for microbial growth and thus less degradation of organic matter. Chen et al. (2022) reported a TOM degradation rate of 12.88% for a co-composting application of SS and food waste, which is similar to this study. With the data attained, it is possible to verify that the degradation with SS was lower, but the overall process seems to be faster.

The pH evolution, shown in Fig. 3A, displays the stabilization in MC after 30 days of composting, although TOM is still not stable. The pH trend of this pile, following the thermophilic phase, remains in the range of 8.0 to 8.5. This ensures an alkaline environment supportive of microbial activities, as highlighted by the TOM trend. In contrast, in the MCS pile, after an initial increase, the pH continuously decreased until the end of the process. Similar trend was observed by Zhang et al. (2023) that was related to the NH_4_^+^-N dynamic, including ammonification and ammonia volatilization during this period. After composting, MC has a higher pH than MCS, 7.97 and 5.78, respectively. The final pH value of MCS is unexpected at the end of a composting process but similar results were obtained by Awasthi et al. (2014), studying a composting process of SS and wheat straw, where a final pH of 5.06 was reported. Numerous studies highlight the influence of pH on metabolic processes during composting. Turan (2008) asserts that pH could be indicative of biological activity, and Sundberg et al. (2004) observed that for pH values near 5, the microbial activity declines.

Figure 3B shows the EC evolution, with higher electrical conductivity in the thermophilic phase for MCS. EC is mainly determined by the organic matter decomposition and mineral fractions content including Na^+^, K^+^, Mg^+^, and HCO_3_^–^. Here, EC tended to be higher in the composting with sewage sludge, which is expected due to the high NH_4_^+^-N and organic acid content caused by the degradation of organic nitrogen and labile precursors enriched in the sewage sludge (Zhang et al. 2023). For both piles, the EC had a high decrease in the beginning, but at the end of the process, both composts have similar EC.

**Fig. 4:**
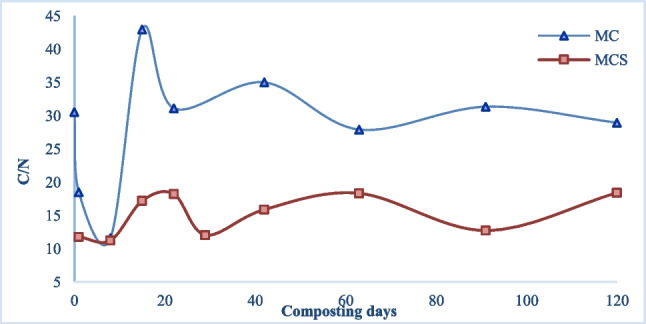
C/N evolution in the composting process

The microbial activity aims to breakdown the organic compounds in order to obtain energy from carbon sources, useful for their metabolism, and acquire several nutrients to sustain their population. Among all, the amount of N is compelling for the composting process, in the cell structure development of the microorganisms. Igoni et al. (2008) outlined the importance of the C/N ratio, showing that a reduced nitrogen content limits the composting process and the microbial activity, resulting in the slow degradation of available carbon. Any excess N above that required by the microbial species, tends to be volatilized as ammonia gas. On the other hand, a low C/N ratio would release conspicuous amounts of basic soluble salts making the compost unfavorable for plant growth (Awasthi et al. 2014). The C/N ratio evolution (Fig. 4) shows a stabilization after around 40 days of composting for MC, consistent with a slowdown in the degradation of organic compounds. Overall, MCS has a final C/N value of 18.4, unlike MC for which C/N is 29.0. It also reaches this stable value after only 29 days of composting. This remarks the influence of SS. The higher contribution of organic nitrogen to the pile by SS, and a difference of only 2.4% in TOC (data not shown), outcomes in a lower C/N ratio than in MC pile, for similar TOC. SS influences not only the C/N ratio at the end of the composting process but also speeds up the maturation of the product, as reported by Chen et al. (2022).

Keeping in mind the application of compost in burned areas for soil restoration, it is very important to evaluate the obtained compost’s quality. A series of physical and chemical analyses were conducted as indicated in Ordinance nº185/2022 (2022), which approves the types of non-harmonized fertilizing materials, defines the type of raw materials that can be used in their production, and provides the requirements for placing them on the market. Many factors determine compost quality, such as waste stream composition, production management, and weather conditions. The physicochemical and biological characteristics and the heavy metal content (e.g., Zn, Cu. Ni, Cr, Pb, and Cd) in the end-product influence the agronomical and environmental performance of systems where compost is used (Quirós et al. 2014). Table 5 shows the physical, chemical, and biological parameters of the three composts produced.

**Table 5:** Composts characterization

Parameters	Compost		
	NMC	MC	MCS
Moisture [%]	60.8 ± 0.3	57.2 ± 1.14	61.8 ± 0.2
TOM [%_dry mass_]	77.7 ± 3.6	53.3 ± 2.2	57.6 ± 1.2
Ashes [%_dry mass_]	22.3 ± 3.6	46.7 ± 2.2	42.4 ± 1.2
TOC [%_dry mass_]	43.2 ± 2.0	29.6 ± 1.2	32.0 ± 0.7
Bulk density [g/cm^3^]	0.44	0.40	0.46
Granulometry [%_pass_]	89.7	92.6	96.8
pH	7.92 ± 0.03	7.97 ± 0.02	5.78 ± 0.02
EC [mS/cm]	0.370 ± 0.017	0.401 ± 0.004	0.444 ± 0.005
TKN [%_dry mass_]	1.00 ± 0.01	1.02 ± 0.02	1.74 ± 0.03
NO_3_^—^N [g/kg_dry mass_]	12.9 ± 0.4	16.9 ± 1.2	76.8 ± 0.7
C/N	43.2	29.0	18.4
Germination index (GI) [%]	146.8	54.5	77.2
Stability class	V	V	V

*EC*, electric conductivity; *TOM*, total organic matter; *TKN*, total Kjeldahl nitrogen.

The two monitored piles (MC and MCS) produced composts with similar quality, considering the TOM, ashes, and TOC. These parameters are considerably lower than the verified for the NMC compost. Moreover, they showed the lower C/N ratio arising from the fact that the organic matter, and therefore the organic carbon, was less degraded in the NMC pile. Nevertheless, the GI reaches considerably high values, notably for the NMC compost. From this data, it is possible to realize that the intervention and pile management are not key factors for the composting of these types of wastes.

Ordinance nº185/2022 (2022) stipulates a maximum moisture content of 40% by weight for compost usage, a value that was exceeded by all the composts studied, particularly for MCS. Comparing with some commercial composts produced with organic fraction of urban wastes, the moisture content is higher. However, moisture content observed in all composts was not very dissimilar from that found by Silva et al. (2009) in a composting application with poultry manure, wood chips, and straw (moisture of 52.6%). This result was achieved without a drying condition process, once it is not the intention to commercialize the compost.

The organic supply added to the soil using compost is helpful in rebuilding the TOM content in soils, mitigating the desertification and erosion phenomena; moreover, the TOC is related to the most stable and slow-release fraction of organic carbon. TOM is higher in NMC compost than in others and exceeds the 30% minimum limit set by the Ordinance nº185/2022 (2022). These results are higher than some commercial composts, whose TOM is around 41%. The main cause of this result may arise from the inefficient aeration of the composted biomass. Without the right amount of oxygen, aerobic bacteria cannot perform their metabolism and respiration, arising anaerobic respiration undermining the decomposition process to slow considerably and the arising of by-products including methane gas and undesirable odors. Another reason for these high TOM values may be related with the time of the process; it seems that 120 days were not enough for the total organic matter degradation. This fact is highlighted by the ashes content that is very low in NMC compost, lower than in MC and MCS, and the high TOM content in each compost: 77.7%_dry mass_, 53.3%_dry mass_, and 57.6%_dry mass_, for NMC, MC, and MCS, respectively. Also, the C/N ratio has similar information, with 43.2, 29.0, and 18.4 for NMC, MC, and MCS, respectively. As expected from the previous observation. TOC presents high values for NMC compost, because, as mentioned above, it was evidently caused by the lack of pile aeration that compromised the metabolic activity of microorganisms and short time for the process of these type of wastes. This confirms the importance of the adequate aeration of compost during the process, promoting a favorable microbial activity and improving the quality of the final product. The C/N ratio found in MCS compost is similar to obtained by implementing a controlled composting process with SS and wheat straw (Awasthi et al. 2014).

The compost’s pH is valuable as it influences the compost’s microbial activity and nutrient availability. Alkaline similar pH value (above 7.9) was found in NMC and MC composts, while MCS has an acidic pH. For all three composts, however, the pH is within the range required by Ordinance (5.5–9) (Ordinance nº185/2022 2022).

The electrical conductivity in a compost is representative of the content of total salts and reflects its quality for use as a soil conditioner (Awasthi et al. 2014; Jiang et al. 2015). It is important to define ways of using compost and its possible effects on crops. The threshold values identified in the scientific literature are distinct. Some studies point out that the EC limit is 4 mS/cm recommended to apply compost to soils (Awasthi et al. 2014; Zhang and Sun 2016; Chowdhury et al. 2015); others estimate a maximum EC value of 2.5 mS/cm (Mulec et al. 2016). However, the values found in the three final composts are below these limits, 0.37 mS/cm for NMC, 0.40 mS/cm for MC, and 0.44 mS/cm for MCS.

The physical characteristics of the compost are also important once impact its application and affect the physical characteristics of the soil. Bulk density is an approximate estimation of sample total porosity; through this value, it is possible to assess the risk of nutrient leaching, soil losses through erosion, and the productivity of agricultural crops. Pampuro et al. (2017) reported that the higher proportion of bigger particles lower the bulk density of a compost. The three composts showed values near the value of 0.4 g/cm^3^, recommended for more mature and stable compost (Zhang and Sun 2016); in this case, no significant effect was observed in relation to pile intervention. Granulometry (%_pass_) is useful to determine the size of the composted material; in this case, none of the composts complies with the legal requirement of more than 99% of the material passing through a sieve of 25 mm, showing that they still have an important amount of agglomerated material that may have difficulty mixing into the soil. However, these values reach for the two monitored piles were higher than NMC, which may indicate that this parameter was influenced by the composting process evolution. These results were associated with the breakdown of AFRs and SS particles during the decomposition of substrate (Fornes et al. 2012). The reduction in particle size to the range between 0.25 and 2 mm increases the water holding capacity, porosity, and apparent density, improving the quality of the organic compost (Zhang and Sun 2016).

The phytotoxicity test to assess the toxicity of the composts was made using the germination index (GI). Composts with GI lower than 60% may have phytotoxic characteristics (Zucconi et al. 1981). GI reaches considerably high values for the NMC compost; this GI value was > 80% (Table 2); according to Zorpas and Loizidou (2008), composts with GI values > 80% are mature and are not phytotoxic. Thus, only NMC compost can be considered mature and stable. The GI value reached for MCS was lower than the value obtained by Zhang et al. (2023).

The maturity level through the self-heating process reached a high maturity level, indicating that conditions were enough to stabilize the organic matter. The three composts showed the highest level of stability (*V*) indicating that the period attained in the process was enough to degrade the biodegradable organic matter. This observation is not in accordance with the high TOM content, fact that prove the needed to assess several quality parameters. Although the quality of final composts was different, all processes reached a relative stable and nonphytotoxic status, demonstrating the feasibility for AFRs composting alone or with sewage sludge.

To complete the compost’s characterization, it is important to assess the nutrients and heavy metal content, to realize the supply of macro and micro nutrients added to the soil, other than potential concentrations of metals harmful to plant species. In terms of nutrient content, the three composts showed similar concentrations (Table 6). Since the EC values of monitored piles at the end of the composting process were similar, the similar nutrient concentrations were expected. According to the Ordinance nº185/2022 (2022), compost is classified into four classes, denoted as I, II, IIA, and III, based on the heavy metal content, with a maximum concentration limit of heavy metals for each class. The heavy metal concentrations analyzed showed that all three composts belonged to class I, as the values found were below the maximum limit for this class of organic corrective. Due to the close values found in nutrient and heavy metal content in NMC and MC, the higher concentration of heavy metals in MCS must arise from SS addition.

**Table 6:** Nutrients and heavy metals presented in composts

	Compost		
	NMC	MC	MCS
Nutrients			
K [%_dry mass_]	0.72 ± 0.02	0.70 ± 0.05	0.58 ± 0.13
Na [%_dry mass_]	0.011 ± 0.002	0.013 ± 0.001	0.016 ± 0.001
Mg [%_dry mass_]	0.28 ± 0.01	0.29 ± 0.02	0.31 ± 0.02
Ca [%_dry mass_]	1.04 ± 0.05	1.27 ± 0.28	0.86 ± 0.09
Heavy metals			
Ni [mg/kg_dry mass_]	3.67 ± 0.59	4.37 ± 0.17	8.05 ± 2.38
Zn [mg/kg_dry mass_]	69.4 ± 3.2	81.2 ± 0.8	145 ± 14
Cd [mg/kg_dry mass_]	0.27 ± 0.02	0.46 ± 0.04	0.60 ± 0.10
Pb [mg/kg_dry mass_]	18.5 ± 4.1	24.0 ± 0.5	22.3 ± 3.7
Cu [mg/kg_dry mass_]	37.4 ± 1.9	38.4 ± 5.3	89.0 ± 5.1
Cr [mg/kg_dry mass_]	21.4 ± 1.5	27.1 ± 4.7	52.6 ± 1.6

The burned land, without organic matter, does not produce lumps, and the soil loses its porosity. In this way rainwater does not penetrate and eventually runs down the surface, grazing fields and pastures, leading to erosion. Without porosity, soil ventilation is hampered, chemical reactions stop, some of the minerals important for nutrition become toxic, plant metabolism becomes slow, and vegetation grows poorly and weak. Under such conditions, even with high fertilization, plant productivity is poor. The soil restoration with stabilized organic wastes used as an amendment, such as the compost, is not only sustainable but is also expected to accelerate the restoration of burned soils ecosystems, not only due to the correction of unbalanced physic-chemical parameters but also because the amendments may serve as inoculant of microorganisms (Vaz-Moreira et al. 2008).

Overall, the composts produced can improve the properties of the soil, since they have high levels of organic matter, are alkaline, promote the plant germination, improving the development of plants, and close the loop of circular economy in the life of agroforestry residues.

### LCA of the composting processes

Due to similar characteristics of MC and NMC composts, shown by the similar composition in nutrients and heavy metals, the impact assessment was performed only for MC and MCS composting processes.

The figures presented below display the contributions (positive or negative) of each process to the total environmental impact of the composting process. Only the concentration of macronutrients (N, P, K) in the composts is considered in the LCA. It disregards considerations concerning compost quality and its actual compliance with legislative values. This aspect is a limitation of the study.

**Fig. 5:**
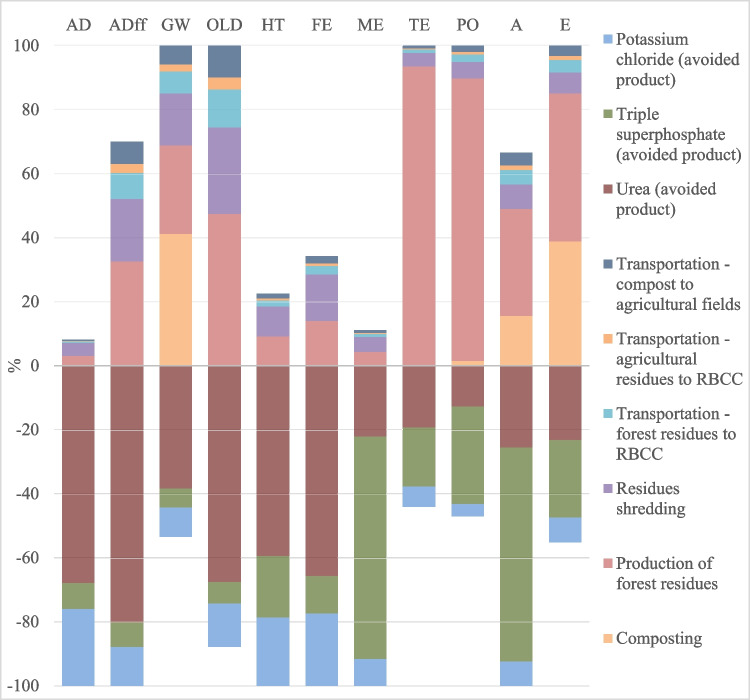
Impact assessment results for composting, MC-abiotic depletion (AD), abiotic depletion associated to fossil fuels (ADff), global warming (GW), ozone layer depletion (OLD), human toxicity (HT), fresh-water aquatic eco-toxicity (FE), marine ecotoxicity (ME), terrestrial ecotoxicity (TE), photochemical oxidation (PO), acidification (A), and eutrophication (E)

Figure 5 shows the LCA for MC composting. Forest residue production in land shows a considerable contribution to all impact categories, especially to terrestrial ecotoxicity (TE) and photochemical oxidation potential (PO), responsible for 88–93% of the impacts. Simultaneously, the shredding of AFRs plays a key role in all categories, with the largest contribution in the ozone layer depletion (OLD) (27%) and abiotic depletion associated to fossil fuels (ADff) (21%) as consequence of the use of oil in the shredding equipment and consequent energetic power for it extraction and production, namely, natural gas and coal, although with lower contribution. The most important factor influencing OLD is Halon 1301 emissions to air. These two categories are also affected by transportation process related impacts (oil, methane, are the most impactful substances). This is in line with the review work of Serafini et al. (2023) that said that the most cited environmental impacts were global warming potential (GW), acidification potential (A), eutrophication potential (E), photochemical oxidation potential (PO), and ozone layer depletion (OLD), as gaseous emissions from the transport and decomposition represent the main contributors to these categories. Composting environmental impacts were also highly related to the use of non-renewable energy sources. Also, Cortés et al. (2020) reported that the consumption of diesel fuel in machinery was determined to be the main critical point in the environmental effects of the system, followed by the transport and distribution of the compost.

The composting process, in which organic matter decomposes and releases emissions, described in the inventory analysis, is shown to be negligible for eight of the eleven impact categories analised. The emission of nitrous oxide strongly impacts on global warning (41%), making the composting process the most impactant on this category. At the same time, acidification represents 16% influence, due to ammonia emissions to air, that also impacts eutrophication, 39% of influence, along with nitrous oxide release. This aspect is in line with Colón et al. (2010), even though a different impact assessment method was used. It was found that ammonia emission contributes for 56% to eutrophication potential and 32% for acidification potential. The eutrophication category is more affected by excess nutrients derived from nitrogen and phosphorus that, in high concentrations in organic waste to be composted, can affect the ecosystem with eutrophication (McBride 2022). Also, Saer et al. (2013) assessed that the decomposition emissions during composting have a large environmental impact on global warming, acidification and eutrophication. Due to oxygen depletion, further emissions can be generated, such as methane, nitrous oxide, and ammonia, which are also greenhouse gases and potential odor sources (Saer et al. 2013; Oliveira et al. 2016).

Considering the consequential approach, in terms of avoided impacts, the avoid urea production contributes more than TSP and potassium chloride for the overall benefits of the composting as soil improvement. The main benefits of its avoided production reflect on abiotic deplection (AD), due to the non-extraction of substances such as tellurium and molybdenum, which can be used in the production of fertilizers, on ADff, due to the avoidance of natural gas, oil, and coal, which are used in the production of energy required for the fertilizer synthesis, and in slightly smaller percentages also on OLD, human toxicity (HT), and fresh-water aquatic eco-toxicity (FE). The triple superphosphate (TSP) is the main contributor to the avoided impacts for TE, PO, A, and E. They are related to the avoided emissions of cypermethrin, very important in TE, sulfur dioxide for PO and A, and phosphorus, leached in water, for E. However, the use of compost on agricultural land only marginally reduces the impacts on global warming, acidification, ecotoxicity, and human toxicity by replacing N, P, and K fertilizers (Zhao and Deng 2014). Cortés et al. (2020) observed that after the application of compost instead of mineral fertilizers, corn, tomato, and strawberry crops would have a better environmental performance in most impact categories, reaching a maximum improvement of 65% in terrestrial ecotoxicity in strawberry cultivation.

**Fig. 6:**
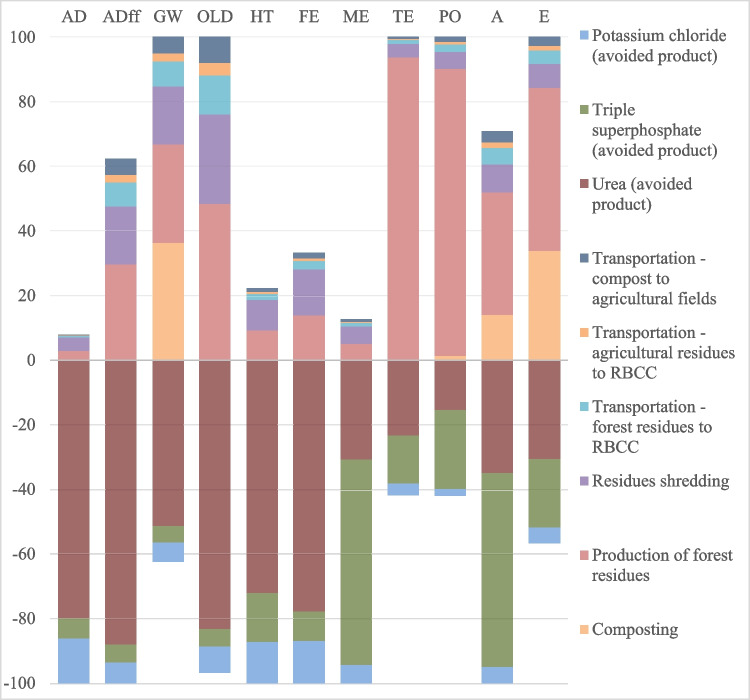
Impact assessment results for composting, MCS-abiotic depletion (AD), abiotic depletion associated to fossil fuels (ADff), global warming (GW), ozone layer depletion (OLD), human toxicity (HT), fresh-water aquatic eco-toxicity (FE), marine ecotoxicity (ME), terrestrial ecotoxicity (TE), photochemical oxidation (PO), acidification (A), and eutrophication (E)

Although, in the MCS composting, the AFRs was mixed with sewage sludge, the results obtained, according to the load allocation criterion used, are very similar to the MC composting examined. The higher nitrogen content of the MCS compost makes the avoided impacts of urea greater than in the previous case. By using a different allocation criterion, the differences may be more pronounced. Results are presented in Fig. 6. Forest residue production has the largest impact for terrestrial ecotoxicity (88%) and contributes 44% to photochemical oxidation. For the other impact categories, its influence is minimal. Ash disposal in landfills contributes to human toxicity (27%), due to the release of substances such as vanadium, nickel, molybdenum, and arsenic into water. The same substances, combined with copper and zinc, also impact on fresh-water aquatic eco-toxicity (63%) and marine ecotoxicity (83%) to a greater extent.

**Fig. 7:**
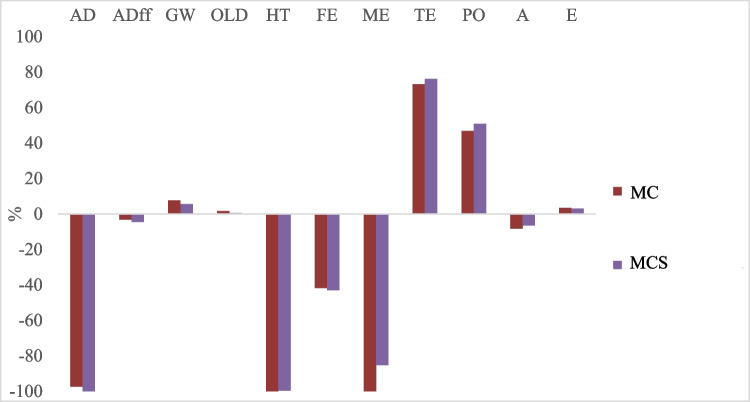
Impact assessment, comparison between two processes—abiotic depletion (AD), abiotic depletion associated to fossil fuels (ADff), global warming (GW), ozone layer depletion (OLD), human toxicity (HT), fresh-water aquatic eco-toxicity (FE), marine ecotoxicity (ME), terrestrial ecotoxicity (TE), photochemical oxidation (PO), acidification (A), and eutrophication (E)

Figure 7 shows the comparation of composting processes to understand which one carries out the highest impact for the categories considered. The two composting processes have a positive impact in abiotic depletion category, as mentioned before, arising from the avoided process of fertilizers raw materials extraction, production, and transportation. The positive impact in terms of acidification (with a negative value) is due to the percentage of nitrogen and phosphorous that is absorbed by the plants and is not released into the soil and water because the selective release of nutrients by compost.

The difference between the two processes is residual, due to the similar characteristics of the biomass subjected to the composting process. It seems that MCS contributes more for the terrestrial ecotoxicity and photochemical oxidation impacts. However, MC composting shows a sligtly higher impact in global warming, ozone layer depletion, and eutrophication than MCS. The quality of the final compost, in terms of nutrients, is the factor that generates the differences between the processes.

### Effects of composts on plant growth

Compost is widely recognized as an organic amendment that has a beneficial impact on the physical, chemical, and biological properties of the soil. Compost promotes the restoration of soil carbon by adding organic matter and the formation of humic substances, thus counteracting the continuous organic matter loss from soils due to agricultural activities (Bong et al. 2017; De Feo et al. 2016). It is also important to note the capacity of compost to be used as a substitute for mineral fertilizers. The effect of compost, MC and MCS, and fertilizer (peat—P) addition to the soil characteristics is shown in Table 7. Overall, the composts improve the soil quality in all the parameters analyzed. The moisture content is similar in soil with additives, showing the improvement in the water capacity holding, and the OM also increased, emphasizing the low OM in the control soil. The same quantity of fertilizer promotes the OM increase more than 2 times in the case of MC and MCS composts and near 4 times with P addition. The differences observed with the three fertilizers are not significant, the main difference is between using peat (P) and compost with stabilized sewage sludges (MCS). The behavior between the two composts from forest wastes are very similar, except for pH and electric conductivity (EC). As discussed previously, the compost with sewage sludges (SS) registered the highest level of minerals, which may increase the EC values.

**Table 7:** Characteristics of the soils with the addition of peat (P) and the AFRs composts (MC and MCS) and the control, with no additives

	Samples			
	Control	Soil + P	Soil + MC	Soil + MCS
pH	5.63 ± 0.00	6.60 ± 0.00	6.93 ± 0.00	5.63 ± 0.00
EC [mS/cm]	0.21 ± 0.02	0.33 ± 0.00	0.42 ± 0.01	1.34 ± 0.13
Moisture [%]	1.00 ± 0.02	15.00 ± 0.78	8.63 ± 4.21	7.97 ± 0.05
OM [%_dry mass_]	3.27 ± 0.03	17.10 ± 0.05	11.32 ± 3.26	12.46 ± 0.14
Ashes [%_dry mass_]	96.7 ± 0.03	82.9 ± 0.05	88.7 ± 3.26	87.5 ± 0.14
TKN [%_dry mass_]	0.09 ± 0.004	0.20 ± 0.000	0.28 ± 0.003	0.39 ± 0.000
CEC [cmole/kg]	1.92 ± 0.022	4.24 ± 0.27	4.26 ± 0.04	4.36 ± 0.06
Mg^2+^[cmole /kg]	0.525	1.06	1.25	1.27
Na^+^[cmole/kg]	0.08	0.20	0.08	0.20
Ca^2+^[cmole /kg]	2.62	5.11	4.01	3.80
K^+^[cmole /kg]	0.08	0.66	0.74	0.08

*EC*, electric conductivity; *OM*, organic matter; *TKN*, total Kjeldahl nitrogen; *CEC*, cation exchangeable capacity.

**Fig. 8:**
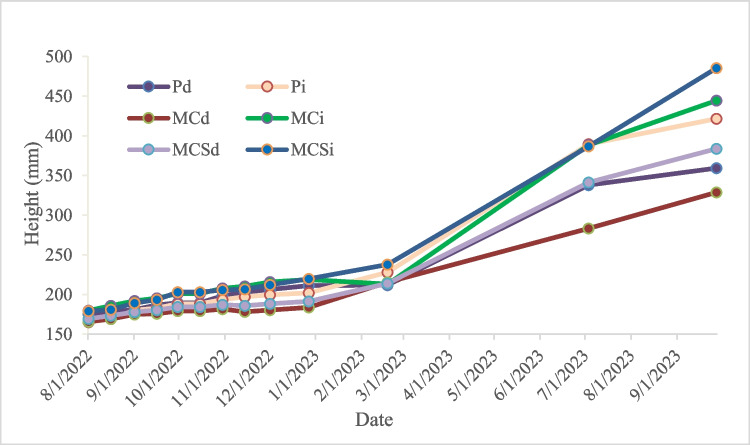
Compost effect on pine growth with (i) and without irrigation (d)

The effect of the compost or peat used in stone pine planting is possible to realize that, regardless of the type of compost used, after the first year of planting, the growth is clearly higher in the treatments with irrigation (i), with the most favorable total growth being obtained with MCSi, followed by MCi, and finally, Pi (Fig. 8). These results show that, under the conditions of this trial, there was an advantage of using AFRs composts as a substitute for the commonly used peat as a support and fertilizer in stone pine plantations. However, if the same composts were used in a situation of water stress or drought (d), the best growth results were again for the MCSd compost, followed by Pd, and finally, MCd, so that, in this case of water stress, only MCSd, but not MCd, achieved better growth than Pd.

It can say that MCS provided greater stone pine growth than MC, which is not surprising given the higher nutritional level of this compost, namely, NO_3_^−^-N. This advantage was more evident in a situation of water stress or drought (d), comparing MCSd with MCd, which leads us to suspect a possible increased moisture retention effect of the MCS. This trend should be evaluated in stone pine future growth.

The chlorophyll fluorescence technique uses chlorophyll in plants as a probe to analyze the effects of various stresses on plants. The measurement of photosynthetic activity (*F*_v_/*F*_m_), the minimum fluorescence (*F*_o_), and the maximum fluorescence (*F*_m_) were made after one year of growth monitorization (Table 8). *F*_v_/*F*_m_ is a parameter widely used to indicate the maximum quantum efficiency of Photosystem II. This parameter is widely considered to be a sensitive indication of plant photosynthetic performance with healthy samples typically achieving a maximum *F*_v_/*F*_m_ value of approximately 0.85. Compared with drought and irrigation trials, *F*_v_/*F*_m_ showed a similar trend, indicating that the light energy conversion efficiency and potential viability in PSII is identical in both situations.

**Table 8:** Photosynthetic activity, *F*_V_/*F*_m_, of the pine leaf after 1 year of trial

Assay	*F*_o_	*F*_m_	*F*_V_/*F*_m_
P_d_	1064 ± 447	2765 ± 911	0.61 ± 0.10
P_i_	1480 ± 856	3631 ± 205	0.60 ± 0.21
MC_d_	1040 ± 173	3675 ± 137	0.72 ± 0.06
MC_i_	1007 ± 278	3465 ± 238	0.71 ± 0.06
MCS_d_	834 ± 43	3515 ± 256	0.76 ± 0.01
MCS_i_	950 ± 118	3393 ± 261	0.72 ± 0.02

The values obtained, lower than 0.85, confirmed that the pines have been exposed to abiotic stress factor (drought) which has reduced the capacity for photochemical quenching of energy within PSII. In this study, the values attained for the photosynthetic activity in drought conditions ranged between 0.61 and 0.76, slight low than the range obtained (0.75 and 0.82) by Balekoglu et al. (2023). However, other stress conditions may be affected the pines because the trials with irrigation registered also values lower than 0.85. Interestingly, the stone pine growth in soil with peat seem to have slightly lower photosynthetic activity, than the others. The MCS compost addition promotes the highest capacity of pines to react to an adverse stress, which is confirmed by the higher growth (Fig. 8). The increase of the richness and diversity of the microorganisms in soil by the compost application (Wang et al. 2024) may explain the plant’s resistance to stress.

## Conclusions

In a broader sense of reducing the risk of wildfires in agroforest areas, this work aims to encourage the valorization of agroforestry residues, comparing qualitative characteristics of three different composting piles, to establish the best practice to adopt. Results show a good quality of unmonitored compost, strengthening the idea of instructing landowners in the practice of composting and compost application, to improve the quality of their own soils. This practice could lead to a management improvement in the waste transferred to the RBCC, as well as an increase in their own soil productivity.

On the other hand, the quality characteristics of MC compost were highlighted. A good practice may be the assembly of smaller piles, promoting better self-aeration. Composting a portion of SS with AFRs does not lead to significant changes in final product quality, compared with compost produced only with AFRs. The environmental impacts of the composting processes, with and without mix of SS, indicated the procedures related with the AFRs production are the main contributing factor, but, mostly, the greater benefit arise from the avoided use of synthetic fertilizers. The main negative environmental impact of composting were the global warming, acidification and eutrophication.

The evolution of soil characteristics after the use of these organic soil conditioners, namely, its effect in stone pine plantation, showed that the soil quality increase, namely, with the improvement of organic matter, carbon and nitrogen content, water retention, and mineral bases, very important for the plant growth processes. Nevertheless, the most important factor evaluated in the plant growth was the irrigation and, consequently, the soil water content, that played an important role in the photosynthetic activity of the plants.

In conclusion, the adequate management of agroforestry residues resulting from pruning and cleaning of green areas involves raising the awareness of those involved to implement waste composting techniques at the production sites. It also can achieve a sustainable strategy of centralized composting, in the RBCC, to allow easy monitoring and distribution, for agricultural soils but also for urban greenery. These practices improve the mitigation of forest wildfires by giving new life to AFRs, enhancing a sustainable waste circular economy. Regardless of the type of compost used, after the first year of stone pine planting, growth is clearly higher in the treatments with irrigation, with the most favorable total growth being obtained with MCS, followed by MC, and finally, P.

## Data Availability

The authors declare that the data supporting the findings of this study are available within the paper.
